# Knockdown of FOXA2 Impairs Hair-Inductive Activity of Cultured Human Follicular Keratinocytes

**DOI:** 10.3389/fcell.2020.575382

**Published:** 2020-10-08

**Authors:** Soon-Sun Bak, Jung Min Park, Ji Won Oh, Jung Chul Kim, Moon Kyu Kim, Young Kwan Sung

**Affiliations:** ^1^Department of Immunology, School of Medicine, Kyungpook National University, Daegu, South Korea; ^2^Department of Anatomy, School of Medicine, Kyungpook National University, Daegu, South Korea; ^3^Clinical Omics Institute, Kyungpook National University, Daegu, South Korea; ^4^Hair Transplantation Center, Kyungpook National University Hospital, Daegu, South Korea

**Keywords:** forkhead box protein A2, hair induction, outer root sheath, transcription factor, trichogenicity

## Abstract

Reciprocal interactions between hair-inductive dermal cells and epidermal cells are essential for *de novo* genesis of hair follicles. Recent studies have shown that outer root sheath (ORS) follicular keratinocytes can be expanded *in vitro*, but the cultured cells often lose receptivity to hair-inducing dermal signals. In this study, we first investigated whether the hair-inductive activity (trichogenicity) of cultured human ORS follicular keratinocytes was correlated with the cultivation period. ORS follicular keratinocytes from the scalp were cultured for 3, 4, 5, or 6 weeks and were then implanted into nude mice along with freshly isolated neonatal mouse dermal cells. We observed that the trichogenicity of the implanted ORS cells was inversely correlated with their cultivation period. These initial findings prompted us to investigate the differentially expressed genes between the short-term (20 days) and long-term (42 days) cultured ORS cells, trichogenic and non-trichogenic, respectively, by microarray analysis. We found that forkhead box protein A2 (FOXA2) was the most up-regulated transcription factor in the trichogenic ORS cells. Thus, we investigated whether the trichogenicity of the cells was affected by FOXA2 expression. We found a significant decrease in the number of induced hair follicles when the ORS cells were transfected with a FOXA2 small interfering RNA versus control small interfering RNA. Taken together, our data strongly suggest that FOXA2 significantly influences the trichogenicity of human ORS cells.

## Introduction

Reciprocal interactive events between the dermal mesenchyme and the overlying epithelium are essential for hair follicle (HF) morphogenesis (Paus and Cotsarelis, 1999; Millar, 2002). Likewise, reciprocal interactions between hair-inductive dermal cells and epithelial cells are needed for *de novo* genesis or neogenesis of HFs (Yang and Cotsarelis, 2010). However, these cells often lose hair-inductive potency (trichogenicity) after conventional *in vitro* culture (Ohyama et al., 2010; Yang and Cotsarelis, 2010). Therefore, to generate HFs by implantation of cells in recipient skin, restoration or maintenance of the trichogenicity of those cells together with expansion of cell numbers is required.

Several strategies have been attempted to restore the trichogenicity of cultured follicular dermal cells, especially dermal papilla (DP) cells, and at least partial restoration was achieved, particularly by spheroid formation and complementation of activators of DP signature pathways (Kang et al., 2012; Ohyama et al., 2012; Higgins et al., 2013; Ohyama and Veraitch, 2013). Regarding epithelial cells for neogenesis of HFs, follicular outer root sheath (ORS) keratinocytes containing hair follicle stem cells (HFSCs) are considered the optimal cells. However, thus far, reports on the restoration of trichogenicity of cultured ORS cells are rare. Chan et al. from Lin’s laboratory have shown that coculture of high-passage rat ORS cells with rat vibrissae DP cells restores trichogenicity (Chan et al., 2015). More recently, we have also shown that the trichogenicity of human ORS cells from occipital scalp can be restored by coculturing with human DP cells (Bak et al., 2018).

In this study, we observed that forkhead box protein A2 (FOXA2), a member of the forkhead/winged-helix family of transcription factors, is the most differentially expressed transcription factor between the short-term and long-term cultured ORS cells by microarray analysis. This, along with inverse correlation of hair-inducing capacity of ORS cells with their cultivation period, led us to investigate whether the trichogenicity of the cells was affected by FOXA2 expression. Using a hair reconstitution assay in combination with a FOXA2 small interfering RNA (siRNA)-mediated knockdown of ORS cells, we show that FOXA2 augments the trichogenicity of cultured human follicular keratinocytes.

## Materials and Methods

### Isolation of Hair Follicles

Biopsy specimens were obtained from non-balding (occipital) scalp of male patients undergoing hair transplantation surgery for androgenetic alopecia. The medical ethical committee of the Kyungpook National University Hospital (Daegu, South Korea) approved all described studies. Written informed consent was obtained from study participants. HFs were isolated using a previously described method with minor modifications (Philpott et al., 1994; Magerl et al., 2002). Briefly, the subcutaneous fat portion of the scalp skin, including the lower HFs, was dissected from the epidermis and dermis. HFs were then isolated under a binocular microscope by forceps.

### Cultivation of Outer Root Sheath Cells

The hair shaft and hair bulb regions of the HFs were cut off to prevent contamination with other cells. The ORS cells were cultured as described before (Kwack et al., 2008). Trimmed hair follicles were immersed in Dulbecco’s modified eagle medium supplemented with 20% fetal bovine serum in Biocoat collagen type I-coated tissue culture dishes (Corning, Kennebunk, ME, United States). On the third day of culture, the medium was changed to EpiLife (Gibco-BRL, Gaithersburg, MD, United States) containing 1% antibiotic-antimycotic solution and 1% EpiLife defined growth supplement. Once subconfluent, the cells were harvested with 0.25% trypsin/10 mM EDTA in phosphate-buffered saline (PBS), split at a 1:5 ratio, and maintained in EpiLife medium. ORS cells in passages 1–4 were used in this study.

### Knockdown of FOXA2 in ORS Cells

The siRNAs used in this study were purchased from Bioneer (Daejeon, South Korea). Cultured human ORS cells were transfected with a control siRNA or a FOXA2 siRNA (Bioneer) at a final concentration of 10 nM in the presence of RNAiMAX reagent (Invitrogen, Carlsbad, CA, United States) for 24 h. Transfected cells (10^4^) were harvested 48 h after transfection and were used for a hair reconstitution assay after the confirmation of a significant knockdown of the genes by real-time polymerase chain reaction (PCR) analysis.

### *In vivo* Patch Hair Reconstitution Assay

Patch hair reconstitution assays were used in this study (Zheng et al., 2005). Dorsal skin from C57BL/6 neonates was collected and incubated overnight with 1 mg/ml collagenase/dispase (Roche, Mannheim, Germany). The dermis and epidermis were separated by incubating the skin with 0.25% trypsin/10 mM EDTA in PBS at 37°C for 15 min. Dermal cells were filtered through 100 μm cell strainers (Becton Dickinson, Franklin, NJ, United States), and then centrifuged at 1,500 rpm for 5 min. Cultured human ORS cells (1 × 10^6^ cells) were combined with freshly isolated neonatal mouse dermal cells (1 × 10^6^ cells) and then subcutaneously implanted into the skin on the backs of 7-week-old female nude mice. After 2 weeks, photos were taken of excised skin samples under a stereoscope, and the reconstituted HFs were quantified. All animal procedures were approved by the Animal Care and Use Committee at Kyungpook National University (Daegu, South Korea).

### Microarrays and Data Analysis

Five micrograms of total RNA from short-term (20 days) and long-term (42 days) ORS cell cultures was used for labeling. Probe synthesis, hybridization, detection, and scanning were performed as described previously (Bak et al., 2018) according to standard protocols from Affymetrix, Inc (Santa Clara, CA, United States). Microarray data were analyzed as we conducted in previous studies (Choi et al., 2017; Wang et al., 2017). Briefly, a bubble chart was used to present biological processes revealed by gene ontology (GO) analysis. The size of circle is dependent on total number of the associated differentially expressed genes (DEGs), while the rank of the circle is set according to *P*-value of analysis. Genes that were significantly associated with these processes were used to compare DEGs with gene set enrichment analysis (GSEA) information related to the skin-associated gene sets as well. Kyoto Encyclopedia of Genes and Genomes (KEGG) 2019 was used for pathway analysis.

### Real-Time PCR

Total RNA was isolated using RNeasy Mini Kit (Qiagen, Austin, TX, United States) according to the manufacturer’s protocol. The purity and integrity of the RNA was measured using a NanoDrop (NanoDrop Technologies, Wilmington, DE, United States). Complementary DNA (cDNA) was synthesized from 3 μg total RNA using a cDNA synthesis kit containing Improm-II reverse transcriptase and oligo-dT primers according to the manufacturer’s instructions (Promega, Madison, WI, United States). Real-time PCR was performed using a StepOnePlus Real-time PCR System (Applied Biosystems, Foster City, CA, United States). All reactions were performed using Power SYBR Green premix (Applied Biosystems), 10 ng cDNA, and 10 μM primers. Amplification was performed under the following cycling conditions: 95°C for 10 min, followed by 40 cycles of 95°C for 15 s and 60°C for 60 s. Primer sequences used were as follows: FOXA2, forward primer 5′-CCCCACAAAATGGACCTCAAG-3′ and reverse primer 5′-GAGTACACCCCCTGGTAGTAG-3′; and GAPDH, forward primer 5′-TGGAAATCCCATCACCATCTTC-3′ and reverse primer 5′-CGCCCCACTTGATTTTGG-3′. Differences between samples and controls were calculated using StepOnePlus Real-Time PCR analysis software (Applied Biosystems).

### Immunocytochemistry

Cultured human ORS cells were plated in each eight-chamber slide (Nunc, Rosklide, Denmark) at a density of 100,000 cells per well. After cultivation in EpiLife medium for 24 h, the chamber slides were fixed with methanol for 20 min at −20°C and equilibrated in PBS for 10 min at 25°C. After blocking with 4% bovine serum albumin (Jackson) for 1 h at 25°C, the slides were incubated with a rabbit polyclonal antibody to SOX9 (1:100 dilution; Abcam) at 4°C overnight, washed four times with PBS, and incubated with donkey anti-rabbit horseradish peroxidase-conjugated antibody (1:100 dilution; Jackson ImmunoResearch, West Grove, PA, United States) for 1 h at 25°C. AEC substrate chromogen (DAKO, Glostrup, Denmark) was used as a color formation reagent for horseradish peroxidase. Finally, slides were counterstained with hematoxylin for 2 min. We used normal rabbit IgG (R&D Systems, Minneapolis, CA, United States) for immunostaining negative controls.

### Statistical Analysis

Data are expressed as mean ± standard deviation (SD). Differences between pairs of groups were analyzed by Student’s *t*-test. A *P-*value of less than 0.05 was considered statistically significant.

## Results and Discussion

**FIGURE 1:**
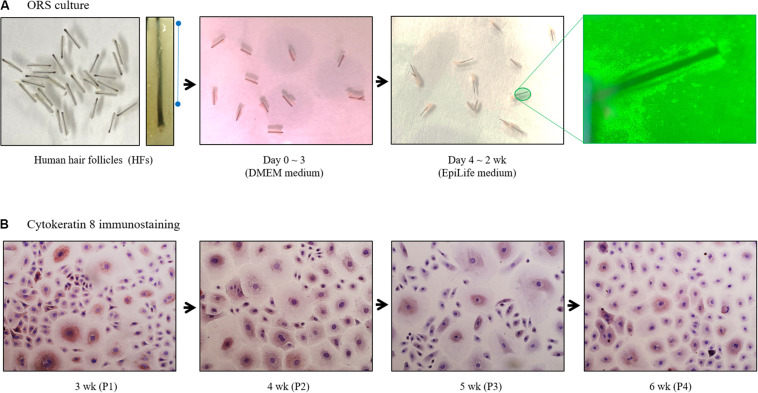
Diagram of human outer root sheath (ORS) follicular keratinocytes cultivation. **(A)** Human ORS cells were cultured as described in the Materials and Methods section. Representative images from a patient (male, age 37 years) are shown. **(B)** Cells in various culture periods (3, 4, 5, and 6 weeks) from a patient (male, age 28 years) were immunostained with cytokeratin 8 antibody.

Trimmed HFs (25–30 HFs/10 cm^2^ dish) were immersed in Dulbecco’s modified eagle medium supplemented with fetal bovine serum in collagen type I-coated tissue culture dishes. Culture medium was then changed to EpiLife medium on the third day of culture (Figure 1A). Once subconfluent, ORS cells were harvested and split at a 1:5 ratio, and maintained in EpiLife medium. Cells were cultured up to 6 weeks and immunostained with cytokeratin 8 antibodies (Figure 1B).

**FIGURE 2:**
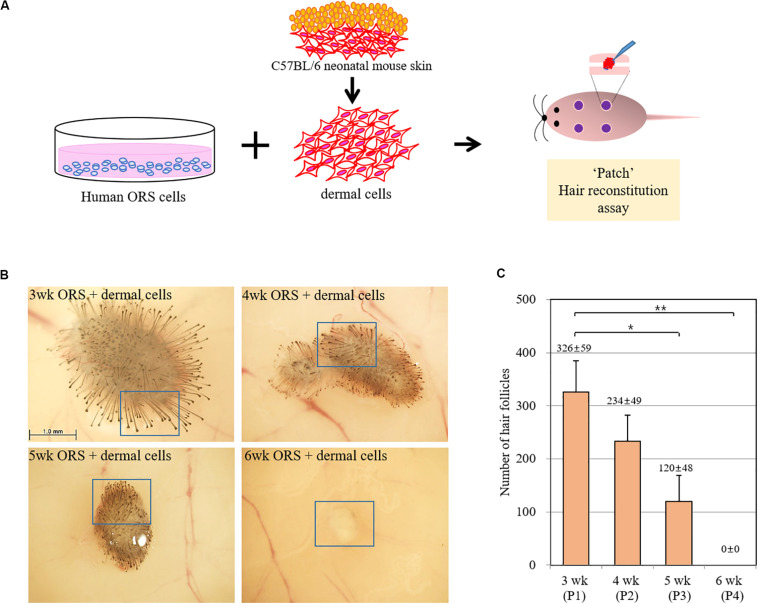
Inverse correlation of hair-inductive activity of ORS cells with their cultivation period. **(A)** Diagram of a “patch” hair reconstitution assay used in this study. Cultured human ORS cells (1 × 10^6^ cells) were combined with freshly isolated neonatal mouse dermal cells (1 × 10^6^ cells) and then subcutaneously implanted into the skin on the backs of 7-week-old female nude mice. **(B)** Hair follicle (HF) formation was examined from the back skin of the mice after 2 weeks and representative pictures are shown. High power images of boxed regions are shown in Supplementary Figure S2. **(C)** Total induced HFs were quantified and the data are the means ± standard deviation of four determinations using same batch ORS cells from a patient (male, age 26 years). *P < 0.05, **P < 0.01.

To investigate whether the hair-inductive activity (trichogenicity) of ORS cells was correlated with the cultivation period, cells in various cultivation periods (3, 4, 5, and 6 weeks) were implanted into nude mice along with freshly isolated neonatal mouse dermal cells (Figure 2A). When quantified, induced hairs were inversely correlated with the cultivation period, and no HF induction was observed in co-implants containing 6-week-old ORS cells and mouse dermal cells (Figures 2B,C and Supplementary Figure S1). The human origin of epithelial cells of reconstituted hair follicles and the mouse origin of mesenchymal part of follicles remains to be proved.

**FIGURE 3:**
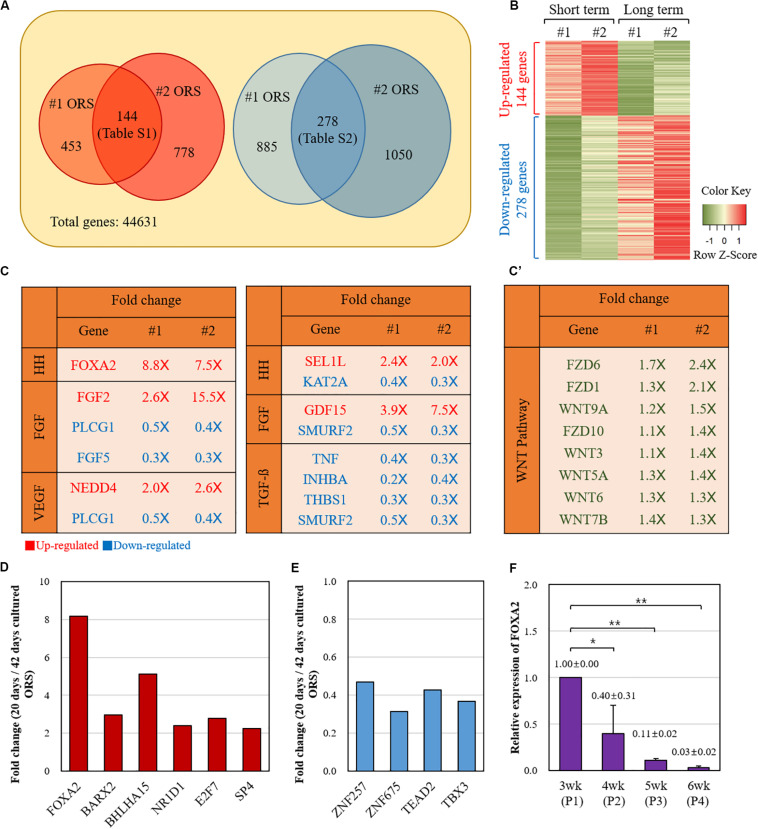
Comparison of the gene expression profiles between the short-term and long-term cultured ORS cells. **(A)** Diagram of twofold up- and down-regulated genes in short-term cultured ORS cells. Two independent cell lines (#1 and #2) from two male patients (ages 50 and 64 years) were cultured for 20 days and 42 days, and microarray analysis was performed with transcripts derived from those cells. The number of genes with a more than twofold increase and decrease in the short-term cultured ORS cells are shown on the left and right, respectively. A total of 144 genes had a more than twofold increase in both short-term cultured ORS cells (listed in Supplementary Table S1), and 278 genes had a more than twofold decrease in both short-term cultured ORS cells (listed in Supplementary Table S2). **(B)** Heat map of differentially expressed genes (DEGs). The up-regulated genes were shown in red color and down-regulated genes in green color. **(C)** List of genes related to the skin signaling pathways from twofold up-regulated (2.0×) and down-regulated (0.5×) genes. Genes were selected based on gene-set enrichment analysis (GSEA) information in each pathway (HH, FGF, VEGF, Notch, BMP, and TGF-b). **(C’)** List of up-regulated Wnts and their receptor (FZD) among WNT signaling pathway. **(D)** Relative fold changes of six up-regulated transcription factors. **(E)** Relative fold changes of four down-regulated transcription factors. **(F)** Relative levels of FOXA2 expression of ORS cells in various cultivation periods (3, 4, 5, and 6 weeks). Data are expressed as means ± standard deviation of three different ORS cell lines from three male patients (ages 33, 38, and 67 years).

Next, we compared the gene expression profiles between the short-term (20 days) and long-term (42 days) cultured ORS cells from two individuals (#1 and #2) by microarray analysis. A number of genes were found to be differentially expressed (Figure 3). Among the 144 genes with a more than twofold increase in short-term cultured ORS cells (Supplementary Table S1), we found 6 transcription factors, including FOXA2, BARX2, and BHLHA15. Also, among the 278 genes with a more than twofold decrease in short-term cultured ORS cells (Supplementary Table S2), we found four transcription factors, including ZNF257and TBX3. Next, we visualized the expression value of DEGs to compare the up-regulated genes and down-regulated genes using Heatmap (Figure 3B) to show the global patterns of the DEGs dynamics between short-term and long-term culture. Then, we used GSEA information to look for the genes that are related to the potential skin associated pathway. Among our findings, FOXA2 is associated with the Hedgehog (HH) signaling pathway, while the other specific genes are related to each pathway (FGF, VEFG, Notch, BMP, TGF-b) (Figure 3C). Intriguingly, FGF5 is highly down-regulated in the short-term culture. At the same time, several genes related to the TGF-b signaling pathway are significantly down-regulated in the short-term culture as well. We also speculated the WNT-related genes between two groups. Total eight genes including WNT7b, WNT9a, WNT3, WNT5a, WNT6, FZD6, FZD1, and FZD10 were identified to be up-regulated, although they are not statistically significant, in WNT signaling pathway (Figure 3C’). To further analysis to find the potential mechanism, the DEGs were used to investigate GO biological process and KEGG pathway (Supplementary Figure S3 and Supplementary Tables S3–S6). The up-regulated genes were enriched in regulation of stem cell proliferation, positive regulation of endothelial cell proliferation, regulation of embryonic development, and negative regulation of cell death. The down-regulated genes were enriched in epidermal cell differentiation, keratinocyte differentiation, and epidermis development (Supplementary Figure S3 and Supplementary Tables S3–S6). The statistically enriched list is summarized in Supplementary Tables S3–S6.

Relative fold changes of the transcription factors, averaged between two individual samples, are shown in Figures 3D,E. We next focused on FOXA2, the most up-regulated transcription factor in the short-term cultured ORS cells compared with long-term cultured ORS cells. In line with microarray data, quantitative reverse transcription-PCR showed that the FOXA2 expression levels gradually decreased as the cultivation period increased (Figure 3F).

**FIGURE 4:**
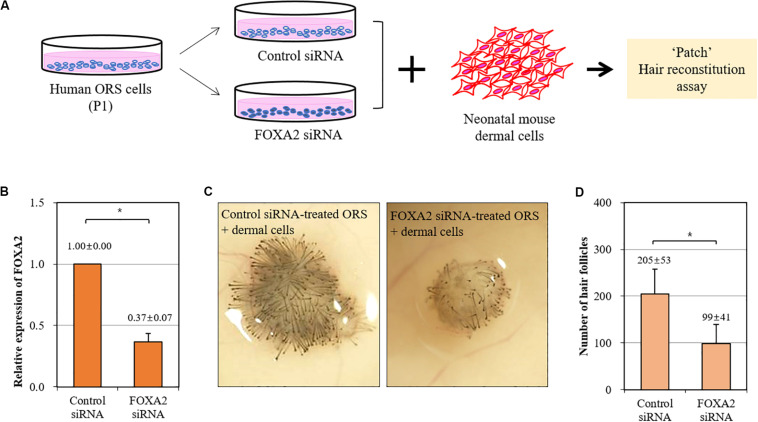
Impairment of hair-inductive activity of ORS cells by knockdown of FOXA2. **(A)** A schematic illustration of the experimental procedure. **(B)** The relative expression of FOXA2 in ORS cells transfected with control siRNA or FOXA2 siRNA was measured by qPCR. **(C)** Representative pictures of induced hair follicles from nude mice skin are shown. **(D)** The total number of induced hair follicles was counted at each injection site. Data are expressed as means ± standard deviation of eight determinations using three different ORS cell lines from three male patients (ages 38, 50, and 58 years). *P < 0.05.

We then investigated whether the trichogenicity of ORS cells was affected by the expression level of FOXA2. We adopted a siRNA-mediated knockdown of FOXA2 in ORS cells in combination with a patch hair reconstitution assay (Figure 4A). Short-term cultured ORS cells were plated for transfection with a FOXA2 siRNA. After transfection for 2 days, the decreased expression of a FOXA2 was confirmed by real-time PCR (Figure 4B). As expected, freshly isolated newborn mouse dermal cells (10^6^ cells) induced HFs when co-transplanted with negative control siRNA-transfected ORS cells (10^6^ cells) (Figure 4C). However, we found a significantly decreased number of induced HFs using FOXA2 siRNA-transfected ORS cells (Figure 4D).

Our data in this study suggest that FOXA2, a transcription factor, may regulate downstream target genes which, in turn, enhance the trichogenicity of ORS cells. Considering that FOXA2 binds to the promoter of WNT7b (Weidenfeld et al., 2002), we propose that FOXA2 might function through WNT7b. Consistent with this hypothesis, we found that the relative fold changes of WNT7b were 1.393 and 1.257 in two individual samples exhibiting 8.840- and 7.506-fold increases of FOXA2, respectively. We speculate that secreted WNT7b from follicular keratinocytes activates inductive dermal cells or makes follicular keratinocytes more susceptible to dermal signals. In this regard, it is interesting to note that loss of WNT7b results in perturbed HF development and differentiation with aberrant hair shaft production (Kandyba and Kobielak, 2014). Overexpression of FOXA2 in cultured ORS cells may help to determine whether this factor can enhance or restore trichogenicity of the cells; these experiments might yield intriguing results and are worthy of further consideration.

## Conclusion

Our data strongly suggest that FOXA2 significantly influences the trichogenicity of human ORS cells. Our findings will provide a rationale for a new strategy for preparing competent human epidermal cells.

## Data Availability Statement

The raw data supporting the conclusions of this article will be made available by the authors, without undue reservation.

## Ethics Statement

The studies involving human participants were reviewed and approved by The Medical Ethical Committee of the Kyungpook National University Hospital (Daegu, South Korea). The patients/participants provided their written informed consent to participate in this study. The animal study was reviewed and approved by the Animal Care and Use Committee at Kyungpook National University (Daegu, South Korea).

## Author Contributions

S-SB and YS designed the research and wrote the manuscript. S-SB performed the experiments. S-SB, JP, and JO analyzed the data. JK and MK contributed the materials that are essential to perform the project. All authors contributed to the article and approved the submitted version.

## Conflict of Interest

The authors declare that the research was conducted in the absence of any commercial or financial relationships that could be construed as a potential conflict of interest.

## Supplementary Material

Supplementary Figure 1 Hair follicles quantified in Figure 2 from the nude mice skin.

Supplementary Figure 2 High power images of boxed regions in Figure 2B.

Supplementary Figure 3 GO analysis and KEGG pathway analysis of microarray data.

Supplementary Table 1 List of genes with a more than twofold increase in short-term cultured ORS cells.

Supplementary Table 2 List of genes with a more than twofold decrease in short-term cultured ORS cells.

Supplementary Table 3 Detail list of up-regulated genes related to GO biological process.

Supplementary Table 4 Detail list of down-regulated genes related to GO biological process.

Supplementary Table 5 Detail list of up-regulated genes related to KEGG pathway.

Supplementary Table 6 Detail list of down-regulated genes related to KEGG pathway.

## Abbreviations

DP: dermal papilla
FOXA2: forkhead box protein A2
HF: hair follicle
ORS: outer root sheath
PBS: phosphate-buffered saline
PCR: polymerase chain reaction
siRNA: small interfering RNA.
