# Supplementary figures and images for: Knockdown of FOXA2 Impairs Hair-Inductive Activity of Cultured Human Follicular Keratinocytes
Source: Front Cell Dev Biol. 2020 Oct 8;8:575382. doi: 10.3389/fcell.2020.575382 (PMC7578224; doi:10.3389/fcell.2020.575382)

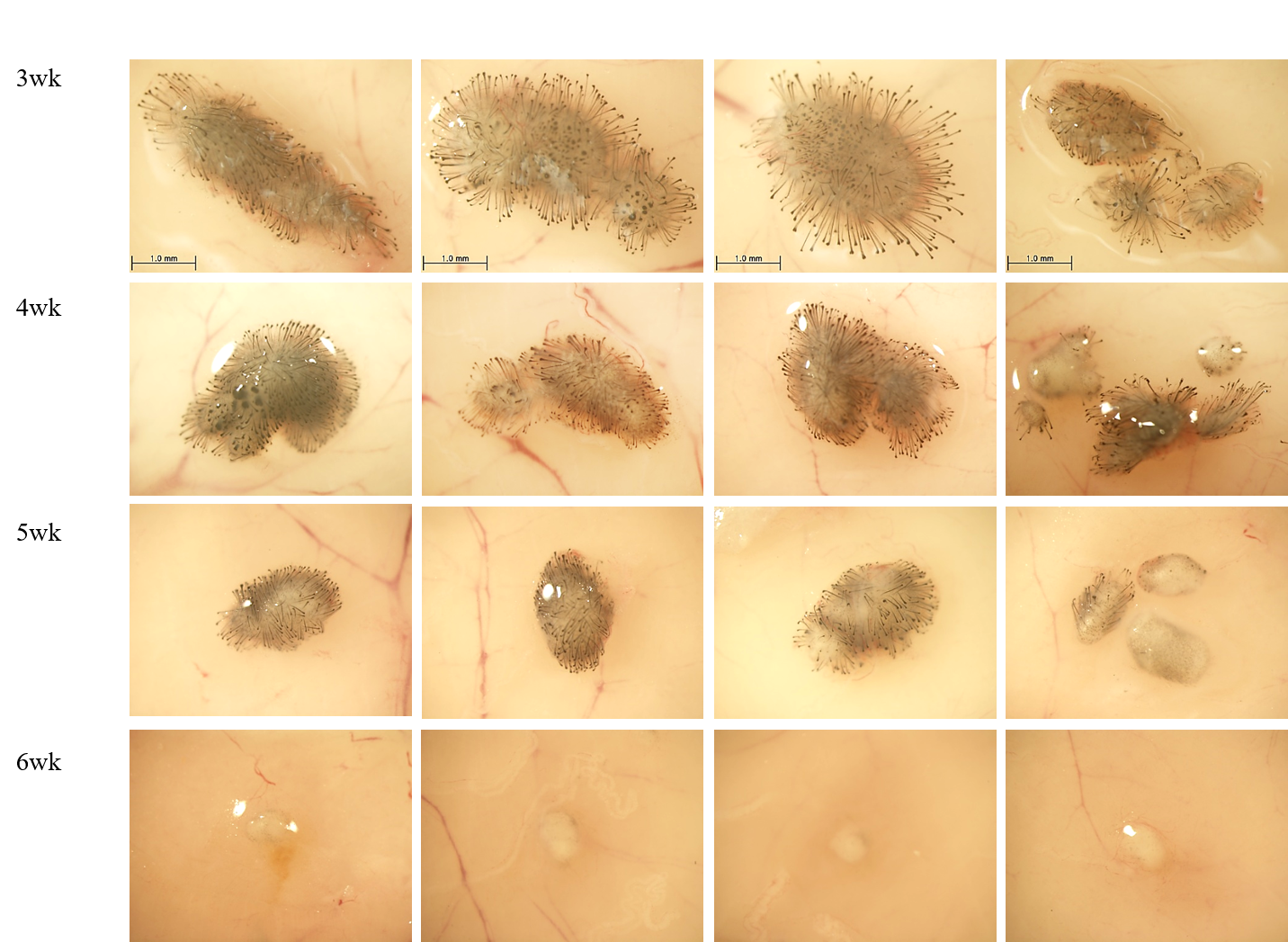

Supplement: Supplementary Figure 1 — Hair follicles quantified in Figure 2 from the nude mice skin. [file Image_1.TIF]

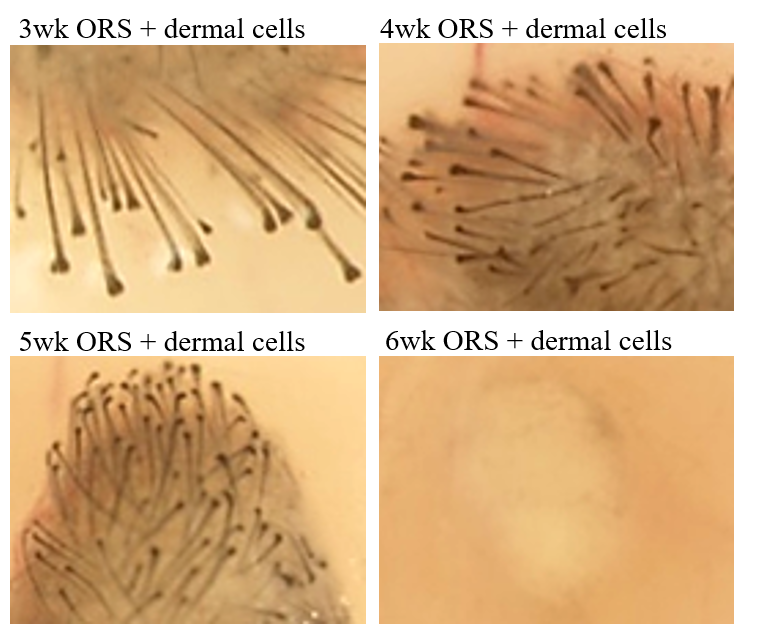

Supplement: Supplementary Figure 2 — High power images of boxed regions in Figure 2B. [file Image_2.TIF]

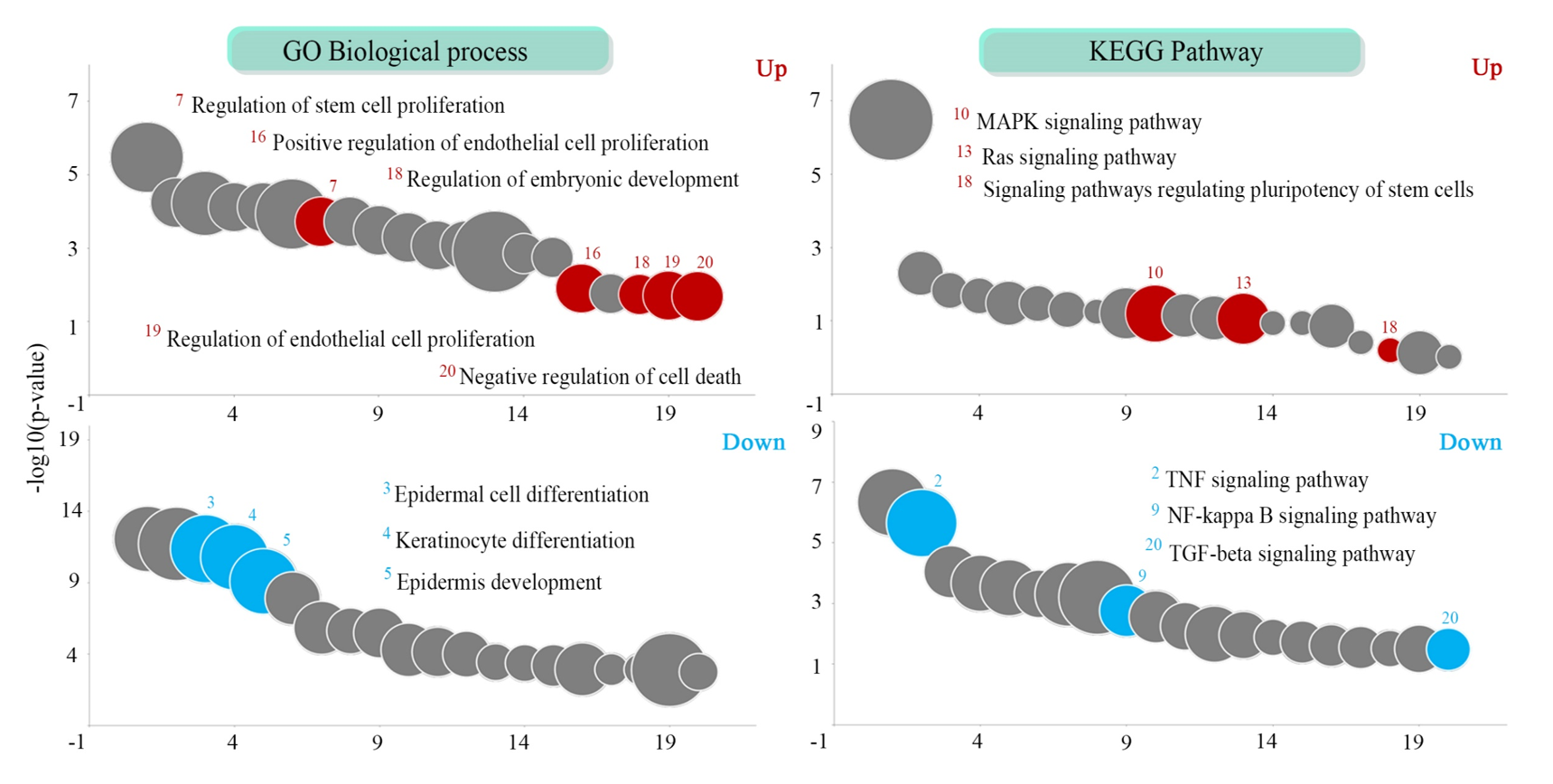

Supplement: Supplementary Figure 3 — GO analysis and KEGG pathway analysis of microarray data. [file Image_3.TIF]
