# Supplementary material for: Knockdown of FOXA2 Impairs Hair-Inductive Activity of Cultured Human Follicular Keratinocytes
Source: Front Cell Dev Biol. 2020 Oct 8;8:575382. doi: 10.3389/fcell.2020.575382 (PMC7578224; doi:10.3389/fcell.2020.575382)
Supplement: Supplementary Table 1 — List of genes with a more than twofold increase in short-term cultured ORS cells. [file Table_1.DOCX]

**Supplementary Table 1. List of genes with a more than 2-fold increase in short-term cultured ORS cells.**

| No. | Gene Symbol | Average Fold change* | Gene Accession | Gene Description |
| --- | --- | --- | --- | --- |
| 1 | FGF2 | 9.079 | NM_002006 | fibroblast growth factor 2 (basic) |
| 2 | FOXA2 | 8.173 | NM_021784 | forkhead box A2 |
| 3 | LAMP3 | 8.056 | NM_014398 | lysosomal-associated membrane protein 3 |
| 4 | CCNA1 | 6.075 | NM_001111045 | cyclin A1 |
| 5 | MBOAT1 | 5.984 | NM_001080480 | membrane bound O-acyltransferase domain containing 1 |
| 6 | GDF15 | 5.698 | NM_004864 | growth differentiation factor 15 |
| 7 | KITLG | 5.672 | NM_000899 | KIT ligand |
| 8 | BHLHA15 | 5.114 | NM_177455 | basic helix-loop-helix family, member a15 |
| 9 | CDK2AP2 | 4.959 | NM_001271849 | cyclin-dependent kinase 2 associated protein 2 |
| 10 | CA5B | 4.837 | ENST00000498004 | carbonic anhydrase VB, mitochondrial |
| 11 | CADPS2 | 4.756 | NM_001009571 | Ca++-dependent secretion activator 2 |
| 12 | RWDD2A | 4.640 | NM_033411 | RWD domain containing 2A |
| 13 | DDIT3 | 4.558 | NM_001195053 | DNA-damage-inducible transcript 3 |
| 14 | CDC6 | 4.425 | NM_001254 | cell division cycle 6 |
| 15 | CCDC163P | 4.250 | NR_033296 | coiled-coil domain containing 163, pseudogene |
| 16 | CLGN | 4.240 | NM_001130675 | calmegin |
| 17 | ERN1 | 4.239 | NM_001433 | endoplasmic reticulum to nucleus signaling 1 |
| 18 | UNC5B | 4.083 | NM_001244889 | unc-5 netrin receptor B |
| 19 | ABHD4 | 3.913 | NM_022060 | abhydrolase domain containing 4 |
| 20 | C6orf48 | 3.849 | NM_001040437 | chromosome 6 open reading frame 48 |
| 21 | NID1 | 3.836 | NM_002508 | nidogen 1 |
| 22 | ARRDC3 | 3.797 | NM_020801 | arrestin domain containing 3 |
| 23 | NGF | 3.783 | NM_002506 | nerve growth factor (beta polypeptide) |
| 24 | SEC11C | 3.717 | NM_001307941 | SEC11 homolog C, signal peptidase complex subunit |
| 25 | LMAN1 | 3.706 | NM_005570 | lectin, mannose-binding, 1 |
| 26 | FIBIN | 3.703 | NM_203371 | fin bud initiation factor homolog (zebrafish) |
| 27 | MYDGF | 3.678 | NM_019107 | myeloid-derived growth factor |
| 28 | DNAJC3 | 3.672 | NM_006260 | DnaJ (Hsp40) homolog, subfamily C, member 3 |
| 29 | HID1 | 3.669 | NM_030630 | HID1 domain containing |
| 30 | HSPA13 | 3.614 | NM_006948 | heat shock protein 70kDa family, member 13 |
| 31 | C4orf32 | 3.561 | NM_152400 | chromosome 4 open reading frame 32 |
| 32 | CATSPER2P1 | 3.545 | NR_002318 | cation channel, sperm associated 2 pseudogene 1 |
| 33 | DNAJB9 | 3.510 | NM_012328 | DnaJ (Hsp40) homolog, subfamily B, member 9 |
| 34 | MIF4GD | 3.490 | NM_001242498 | MIF4G domain containing |
| 35 | ARG2 | 3.476 | NM_001172 | arginase 2 |
| 36 | ABI3BP | 3.389 | NM_015429 | ABI family, member 3 (NESH) binding protein |
| 37 | PARM1 | 3.385 | NM_015393 | prostate androgen-regulated mucin-like protein 1 |
| 38 | ERO1B | 3.383 | NM_019891 | endoplasmic reticulum oxidoreductase beta |
| 39 | CTBS | 3.306 | NM_004388 | chitobiase, di-N-acetyl- |
| 40 | PER3 | 3.269 | NM_001289861 | period circadian clock 3 |
| 41 | SLC7A11 | 3.237 | NM_014331 | solute carrier family 7 (anionic amino acid transporter light chain, xc- system), member 11 |
| 42 | ALDH6A1 | 3.223 | NM_001278593 | aldehyde dehydrogenase 6 family, member A1 |
| 43 | BLNK | 3.188 | NM_001114094 | B-cell linker |
| 44 | ERVE-1 | 3.166 | BC037342 | endogenous retrovirus group E, member 1 |
| 45 | PEG10 | 3.149 | NM_001040152 | paternally expressed 10 |
| 46 | SLC22A3 | 3.137 | NM_021977 | solute carrier family 22 (organic cation transporter), member 3 |
| 47 | H19 | 3.107 | NR_002196 | H19, imprinted maternally expressed transcript (non-protein coding) |
| 48 | GPCPD1 | 3.096 | NM_019593 | glycerophosphocholine phosphodiesterase 1 |
| 49 | BCAM | 3.080 | NM_001013257 | basal cell adhesion molecule (Lutheran blood group) |
| 50 | RAB27A | 3.070 | NM_004580 | RAB27A, member RAS oncogene family |
| 51 | GPNMB | 3.070 | NM_001005340 | glycoprotein (transmembrane) nmb |
| 52 | LOC105369502 | 3.061 | XR_913542 | uncharacterized LOC105369502 |
| 53 | NUCB2 | 3.018 | NM_005013 | nucleobindin 2 |
| 54 | PITPNM3 | 2.990 | NM_001165966 | PITPNM family member 3 |
| 55 | BARX2 | 2.971 | NM_003658 | BARX homeobox 2 |
| 56 | SEC23B | 2.956 | NM_001172745 | Sec23 homolog B, COPII coat complex component |
| 57 | WFS1 | 2.928 | NM_001145853 | Wolfram syndrome 1 (wolframin) |
| 58 | ATP6V0D2 | 2.905 | NM_152565 | ATPase, H+ transporting, lysosomal 38kDa, V0 subunit d2 |
| 59 | SGCG | 2.893 | NM_000231 | sarcoglycan gamma |
| 60 | ZBTB8A | 2.888 | NM_001040441 | zinc finger and BTB domain containing 8A |
| 61 | CYP39A1 | 2.877 | NM_001278738 | cytochrome P450, family 39, subfamily A, polypeptide 1 |
| 62 | UNC5B-AS1 | 2.875 | NR_038453 | UNC5B antisense RNA 1 |
| 63 | ARFGAP3 | 2.874 | NM_001142293 | ADP-ribosylation factor GTPase activating protein 3 |
| 64 | CERS3 | 2.838 | NM_001290341 | ceramide synthase 3 |
| 65 | HIST1H2BN | 2.826 | ENST00000449538 | histone cluster 1, H2bn |
| 66 | SLC50A1 | 2.796 | NM_001122837 | solute carrier family 50 (sugar efflux transporter), member 1 |
| 67 | LARP1B | 2.782 | NM_001278604 | La ribonucleoprotein domain family, member 1B |
| 68 | E2F7 | 2.782 | NM_203394 | E2F transcription factor 7 |
| 69 | FAM46A | 2.781 | NM_017633 | family with sequence similarity 46, member A |
| 70 | CYP3A5 | 2.765 | NM_000777 | cytochrome P450, family 3, subfamily A, polypeptide 5 |
| 71 | FKBP5 | 2.743 | NM_001145775 | FK506 binding protein 5 |
| 72 | UPP1 | 2.716 | NM_001287426 | uridine phosphorylase 1 |
| 73 | SLC10A7 | 2.700 | NM_001029998 | solute carrier family 10, member 7 |
| 74 | LOC105378653 | 2.684 | XR_947200 | uncharacterized LOC105378653 |
| 75 | MIR22HG | 2.683 | NR_028502 | MIR22 host gene |
| 76 | ALPK1 | 2.655 | NM_001102406 | alpha kinase 1 |
| 77 | RGS16 | 2.651 | NM_002928 | regulator of G-protein signaling 16 |
| 78 | ERICH2 | 2.620 | NM_001290030 | glutamate rich 2 |
| 79 | TULP3 | 2.602 | NM_001160408 | tubby like protein 3 |
| 80 | CYB561 | 2.601 | NM_001017916 | cytochrome b561 |
| 81 | ANAPC16 | 2.598 | NM_001242546 | anaphase promoting complex subunit 16 |
| 82 | FRRS1 | 2.591 | NM_001013660 | ferric-chelate reductase 1 |
| 83 | SAT2 | 2.589 | NM_133491 | spermidine/spermine N1-acetyltransferase family member 2 |
| 84 | MIR3665 | 2.573 | NR_037438 | microRNA 3665 |
| 85 | SMIM14 | 2.549 | NM_174921 | small integral membrane protein 14 |
| 86 | CCNB1IP1 | 2.541 | NM_021178 | cyclin B1 interacting protein 1, E3 ubiquitin protein ligase |
| 87 | SLC16A7 | 2.538 | NM_001270622 | solute carrier family 16 (monocarboxylate transporter), member 7 |
| 88 | AMN1 | 2.518 | NM_001113402 | antagonist of mitotic exit network 1 homolog |
| 89 | GFPT1 | 2.516 | NM_001244710 | glutamine--fructose-6-phosphate transaminase 1 |
| 90 | HMGB2 | 2.511 | NM_001130688 | high mobility group box 2 |
| 91 | EPCAM | 2.474 | NM_002354 | epithelial cell adhesion molecule |
| 92 | SMDT1 | 2.460 | NM_033318 | single-pass membrane protein with aspartate-rich tail 1 |
| 93 | GOLPH3L | 2.456 | NM_018178 | golgi phosphoprotein 3-like |
| 94 | SPACA5 | 2.454 | uc004diu.3 | sperm acrosome associated 5 |
| 95 | SMOC1 | 2.433 | NM_001034852 | SPARC related modular calcium binding 1 |
| 96 | SNHG17 | 2.415 | NR_015366 | small nucleolar RNA host gene 17 |
| 97 | DNAJC27 | 2.411 | NM_001198559 | DnaJ (Hsp40) homolog, subfamily C, member 27 |
| 98 | HIST2H2AB | 2.410 | NM_175065 | histone cluster 2, H2ab |
| 99 | ZBED3 | 2.409 | NM_032367 | zinc finger, BED-type containing 3 |
| 100 | AMZ2 | 2.394 | NM_001033569 | archaelysin family metallopeptidase 2 |
| 101 | NR1D1 | 2.393 | NM_021724 | nuclear receptor subfamily 1, group D, member 1 |
| 102 | UAP1L1 | 2.386 | NM_207309 | UDP-N-acetylglucosamine pyrophosphorylase 1 like 1 |
| 103 | SNHG8 | 2.378 | NR_003584 | small nucleolar RNA host gene 8 |
| 104 | BLVRB | 2.377 | NM_000713 | biliverdin reductase B |
| 105 | ZNF14 | 2.375 | NM_021030 | zinc finger protein 14 |
| 106 | PGM3 | 2.371 | NM_001199917 | phosphoglucomutase 3 |
| 107 | GOLT1A | 2.357 | NM_198447 | golgi transport 1A |
| 108 | SGTB | 2.351 | NM_019072 | small glutamine-rich tetratricopeptide repeat (TPR)-containing, beta |
| 109 | TMEM47 | 2.328 | NM_031442 | transmembrane protein 47 |
| 110 | TMEM116 | 2.326 | NM_001193453 | transmembrane protein 116 |
| 111 | NEDD4 | 2.324 | NM_001284338 | neural precursor cell expressed, developmentally down-regulated 4, E3 ubiquitin protein ligase |
| 112 | MIS12 | 2.323 | NM_001258217 | MIS12 kinetochore complex component |
| 113 | DNMT3B | 2.323 | NM_001207055 | DNA (cytosine-5-)-methyltransferase 3 beta |
| 114 | LYSMD3 | 2.320 | NM_001286812 | LysM, putative peptidoglycan-binding, domain containing 3 |
| 115 | UGDH-AS1 | 2.307 | NR_047679 | UGDH antisense RNA 1 |
| 116 | HIST1H3I | 2.304 | NM_003533 | histone cluster 1, H3i |
| 117 | ZFAS1 | 2.294 | NR_003604 | ZNFX1 antisense RNA 1 |
| 118 | CLEC18A | 2.288 | NM_001136214 | C-type lectin domain family 18, member A |
| 119 | RPS6KA6 | 2.276 | NM_014496 | ribosomal protein S6 kinase, 90kDa, polypeptide 6 |
| 120 | APTR | 2.275 | NR_038361 | Alu-mediated CDKN1A/p21 transcriptional regulator (non-protein coding) |
| 121 | SP4 | 2.262 | NM_003112 | Sp4 transcription factor |
| 122 | ERLEC1 | 2.260 | NM_001127397 | endoplasmic reticulum lectin 1 |
| 123 | PLA2G12A | 2.256 | NM_030821 | phospholipase A2, group XIIA |
| 124 | P2RY1 | 2.255 | NM_002563 | purinergic receptor P2Y, G-protein coupled, 1 |
| 125 | ARMCX3 | 2.254 | NM_016607 | armadillo repeat containing, X-linked 3 |
| 126 | DERL2 | 2.250 | NM_001304777 | derlin 2 |
| 127 | PRPSAP2 | 2.236 | NM_001243936 | phosphoribosyl pyrophosphate synthetase-associated protein 2 |
| 128 | ZC3H6 | 2.232 | NM_198581 | zinc finger CCCH-type containing 6 |
| 129 | COBLL1 | 2.229 | NM_001278458 | cordon-bleu WH2 repeat protein like 1 |
| 130 | SEL1L | 2.219 | NM_001244984 | sel-1 suppressor of lin-12-like (C. elegans) |
| 131 | ARHGAP42 | 2.215 | NM_152432 | Rho GTPase activating protein 42 |
| 132 | EAF2 | 2.211 | NM_018456 | ELL associated factor 2 |
| 133 | ARL13B | 2.194 | NM_001174150 | ADP-ribosylation factor like GTPase 13B |
| 134 | RAB33B | 2.192 | NM_031296 | RAB33B, member RAS oncogene family |
| 135 | CD274 | 2.189 | NM_001267706 | CD274 molecule |
| 136 | RPS15A | 2.178 | NM_001019 | ribosomal protein S15a |
| 137 | SHISA4 | 2.176 | NM_198149 | shisa family member 4 |
| 138 | NFXL1 | 2.153 | NM_001278623 | nuclear transcription factor, X-box binding-like 1 |
| 139 | KIAA0226L | 2.145 | NM_001286761 | KIAA0226-like |
| 140 | LIN52 | 2.139 | NM_001024674 | lin-52 DREAM MuvB core complex component |
| 141 | FBLN1 | 2.138 | NM_001996 | fibulin 1 |
| 142 | CORO2A | 2.115 | NM_003389 | coronin, actin binding protein, 2A |
| 143 | MARVELD2 | 2.103 | NM_001038603 | MARVEL domain containing 2 |
| 144 | PAN2 | 2.081 | NM_001127460 | PAN2 poly(A) specific ribonuclease subunit |

* The value applies the fold change which compares short-term and long-term cultured samples. It is averaged between two individual samples that were separately experimented.
