# Supplementary material for: Knockdown of FOXA2 Impairs Hair-Inductive Activity of Cultured Human Follicular Keratinocytes
Source: Front Cell Dev Biol. 2020 Oct 8;8:575382. doi: 10.3389/fcell.2020.575382 (PMC7578224; doi:10.3389/fcell.2020.575382)
Supplement: Supplementary Table 2 — List of genes with a more than twofold decrease in short-term cultured ORS cells. [file Table_2.DOCX]

**Supplementary Table 2. List of genes with a more than 2-fold decrease in short-term cultured ORS cells.**

| No. | Gene Symbol | Average Fold change* | Gene Accession | Gene Description |
| --- | --- | --- | --- | --- |
| 1 | SFTA1P | 0.492 | NR_027082 | surfactant associated 1, pseudogene |
| 2 | MT2A | 0.482 | NM_005953 | metallothionein 2A |
| 3 | FANK1 | 0.481 | NM_145235 | fibronectin type III and ankyrin repeat domains 1 |
| 4 | EPGN | 0.480 | NM_001270989 | epithelial mitogen |
| 5 | KNSTRN | 0.479 | NM_001142761 | kinetochore-localized astrin/SPAG5 binding protein |
| 6 | MARVELD1 | 0.479 | NM_031484 | MARVEL domain containing 1 |
| 7 | RPGRIP1L | 0.479 | NM_001127897 | RPGRIP1-like |
| 8 | CCNE1 | 0.475 | NM_001238 | cyclin E1 |
| 9 | ZNF257 | 0.470 | NM_033468 | zinc finger protein 257 |
| 10 | PPP4R4 | 0.469 | NM_020958 | protein phosphatase 4, regulatory subunit 4 |
| 11 | ITGB4 | 0.469 | NM_000213 | integrin beta 4 |
| 12 | LOC101927123 | 0.462 | NR_110147 | uncharacterized LOC101927123 |
| 13 | NEIL1 | 0.462 | NM_001256552 | nei-like DNA glycosylase 1 |
| 14 | SNORA2C | 0.460 | NR_002968 | small nucleolar RNA, H/ACA box 2C |
| 15 | SNORD89 | 0.460 | NR_003070 | small nucleolar RNA, C/D box 89 |
| 16 | GJB2 | 0.457 | NM_004004 | gap junction protein beta 2 |
| 17 | SNORD116-26 | 0.453 | NR_003340 | small nucleolar RNA, C/D box 116-26 |
| 18 | LOC105370777 | 0.452 | XR_916226 | uncharacterized LOC105370777 |
| 19 | SNRNP70 | 0.451 | NM_001301069 | small nuclear ribonucleoprotein, U1 70kDa subunit |
| 20 | CSMD3 | 0.448 | NM_052900 | CUB and Sushi multiple domains 3 |
| 21 | HNRNPA3 | 0.448 | NM_194247 | heterogeneous nuclear ribonucleoprotein A3 |
| 22 | PLCG1 | 0.446 | NM_002660 | phospholipase C, gamma 1 |
| 23 | NFKBIZ | 0.442 | NM_001005474 | nuclear factor of kappa light polypeptide gene enhancer in B-cells inhibitor, zeta |
| 24 | ADGRA3 | 0.442 | NM_145290 | adhesion G protein-coupled receptor A3 |
| 25 | DRAM1 | 0.442 | NM_018370 | DNA-damage regulated autophagy modulator 1 |
| 26 | LINC00704 | 0.440 | NR_024475 | long intergenic non-protein coding RNA 704 |
| 27 | HSPA1A | 0.439 | NM_005345 | heat shock 70kDa protein 1A |
| 28 | SLC37A1 | 0.438 | NM_018964 | solute carrier family 37 (glucose-6-phosphate transporter), member 1 |
| 29 | SNORD127 | 0.435 | NR_003691 | small nucleolar RNA, C/D box 127 |
| 30 | TEAD2 | 0.427 | NM_001256658 | TEA domain family member 2 |
| 31 | SNORA8 | 0.427 | NR_002920 | small nucleolar RNA, H/ACA box 8 |
| 32 | SPINK1 | 0.426 | NM_003122 | serine peptidase inhibitor, Kazal type 1 |
| 33 | CARD6 | 0.425 | NM_032587 | caspase recruitment domain family, member 6 |
| 34 | SETBP1 | 0.422 | NM_001130110 | SET binding protein 1 |
| 35 | TNF | 0.422 | NM_000594 | tumor necrosis factor |
| 36 | SNORD8 | 0.421 | NR_002916 | small nucleolar RNA, C/D box 8 |
| 37 | FJX1 | 0.417 | NM_014344 | four jointed box 1 |
| 38 | ZNF737 | 0.416 | NM_001159293 | zinc finger protein 737 |
| 39 | SNAR-H | 0.415 | NR_024342 | small ILF3/NF90-associated RNA H |
| 40 | GANC | 0.413 | NM_001301409 | glucosidase, alpha; neutral C |
| 41 | SMURF2 | 0.412 | NM_022739 | SMAD specific E3 ubiquitin protein ligase 2 |
| 42 | SNORD91B | 0.409 | NR_003073 | small nucleolar RNA, C/D box 91B |
| 43 | LOC100288778 | 0.409 | NR_130745 | WAS protein family homolog 1 pseudogene |
| 44 | IGSF3 | 0.408 | NM_001007237 | immunoglobulin superfamily, member 3 |
| 45 | HIST1H2BC | 0.407 | ENST00000314332 | histone cluster 1, H2bc |
| 46 | GPAT4 | 0.407 | NM_178819 | glycerol-3-phosphate acyltransferase 4 |
| 47 | ZBED2 | 0.405 | NM_024508 | zinc finger, BED-type containing 2 |
| 48 | LOC105373148 | 0.400 | XR_921264 | uncharacterized LOC105373148 |
| 49 | KAT8 | 0.399 | XM_011545969 | K(lysine) acetyltransferase 8 |
| 50 | SNORD14E | 0.399 | NR_003125 | small nucleolar RNA, C/D box 14E |
| 51 | ITGA2 | 0.398 | NM_002203 | integrin, alpha 2 (CD49B, alpha 2 subunit of VLA-2 receptor) |
| 52 | SCARNA21 | 0.397 | NR_003000 | small Cajal body-specific RNA 21 |
| 53 | SNRPN | 0.397 | AF400487 | small nuclear ribonucleoprotein polypeptide N |
| 54 | FANCM | 0.396 | NM_001308133 | Fanconi anemia complementation group M |
| 55 | MIR4668 | 0.396 | NR_039814 | microRNA 4668 |
| 56 | MT1X | 0.393 | NM_005952 | metallothionein 1X |
| 57 | HIST2H2BC | 0.392 | NR_036461 | histone cluster 2, H2bc (pseudogene) |
| 58 | GLA | 0.390 | NM_000169 | galactosidase, alpha |
| 59 | CENPT | 0.389 | NM_025082 | centromere protein T |
| 60 | NOP56 | 0.389 | NM_006392 | NOP56 ribonucleoprotein |
| 61 | MAP3K8 | 0.388 | XM_011519310 | mitogen-activated protein kinase kinase kinase 8 |
| 62 | PGGT1B | 0.388 | XM_005272020 | protein geranylgeranyltransferase type I, beta subunit |
| 63 | KPNA2 | 0.387 | NM_002266 | karyopherin alpha 2 (RAG cohort 1, importin alpha 1) |
| 64 | TRIML2 | 0.387 | NM_001303419 | tripartite motif family like 2 |
| 65 | MIR3916 | 0.385 | NR_037480 | microRNA 3916 |
| 66 | SNORD41 | 0.384 | NR_002751 | small nucleolar RNA, C/D box 41 |
| 67 | IPO5P1 | 0.384 | NR_103741 | importin 5 pseudogene 1 |
| 68 | SYT14 | 0.381 | NM_001146261 | synaptotagmin XIV |
| 69 | PLEKHM1P | 0.381 | OTTHUMT00000445597 | pleckstrin homology domain containing, family M (with RUN domain) member 1 pseudogene |
| 70 | SNORA16B | 0.381 | NR_004389 | small nucleolar RNA, H/ACA box 16B |
| 71 | LOC440300 | 0.380 | NR_033738 | chondroitin sulfate proteoglycan 4 pseudogene |
| 72 | MIR554 | 0.379 | NR_030280 | microRNA 554 |
| 73 | KIAA1549L | 0.378 | NM_012194 | KIAA1549-like |
| 74 | STC1 | 0.376 | NM_003155 | stanniocalcin 1 |
| 75 | C20orf96 | 0.376 | NM_080571 | chromosome 20 open reading frame 96 |
| 76 | LOC101927686 | 0.374 | NR_125844 | uncharacterized LOC101927686 |
| 77 | BICC1 | 0.374 | NM_001080512 | BicC family RNA binding protein 1 |
| 78 | SNRPN | 0.374 | AF400486 | small nuclear ribonucleoprotein polypeptide N |
| 79 | TNFSF10 | 0.373 | NM_001190942 | tumor necrosis factor (ligand) superfamily, member 10 |
| 80 | SNORA12 | 0.372 | NR_002954 | small nucleolar RNA, H/ACA box 12 |
| 81 | KAT2A | 0.372 | NM_021078 | K(lysine) acetyltransferase 2A |
| 82 | IGFL3 | 0.371 | NM_207393 | IGF like family member 3 |
| 83 | NUAK1 | 0.371 | NM_014840 | NUAK family, SNF1-like kinase, 1 |
| 84 | SNORA71B | 0.370 | NR_002910 | small nucleolar RNA, H/ACA box 71B |
| 85 | SNORA49 | 0.370 | NR_002979 | small nucleolar RNA, H/ACA box 49 |
| 86 | RNVU1-14 | 0.369 | NR_104075 | RNA, variant U1 small nuclear 14 |
| 87 | FBXL19-AS1 | 0.368 | NR_024348 | FBXL19 antisense RNA 1 (head to head) |
| 88 | TBX3 | 0.367 | NM_005996 | T-box 3 |
| 89 | MIR24-2 | 0.367 | NR_029497 | microRNA 24-2 |
| 90 | CEACAM5 | 0.366 | NM_001291484 | carcinoembryonic antigen-related cell adhesion molecule 5 |
| 91 | PGM2L1 | 0.365 | NM_173582 | phosphoglucomutase 2-like 1 |
| 92 | ERV3-1 | 0.364 | NM_001007253 | endogenous retrovirus group 3, member 1 |
| 93 | ZNF724P | 0.363 | NR_045525 | zinc finger protein 724, pseudogene |
| 94 | SNORD103A | 0.361 | NR_004054 | small nucleolar RNA, C/D box 103A |
| 95 | SNORD103A | 0.361 | NR_004054 | small nucleolar RNA, C/D box 103A |
| 96 | KCTD11 | 0.359 | NM_001002914 | potassium channel tetramerization domain containing 11 |
| 97 | SNAR-C3 | 0.359 | NR_024221 | small ILF3/NF90-associated RNA C3 |
| 98 | SNAR-C4 | 0.359 | NR_024218 | small ILF3/NF90-associated RNA C4 |
| 99 | SEMA7A | 0.357 | NM_001146029 | semaphorin 7A, GPI membrane anchor (John Milton Hagen blood group) |
| 100 | SAA1 | 0.356 | NM_000331 | serum amyloid A1 |
| 101 | MT1B | 0.356 | NM_005947 | metallothionein 1B |
| 102 | SNORA2A | 0.355 | NR_002950 | small nucleolar RNA, H/ACA box 2A |
| 103 | SNORD101 | 0.354 | NR_002434 | small nucleolar RNA, C/D box 101 |
| 104 | LINC01204 | 0.352 | NR_104644 | long intergenic non-protein coding RNA 1204 |
| 105 | SNORD35B | 0.352 | NR_001285 | small nucleolar RNA, C/D box 35B |
| 106 | IQCA1 | 0.351 | NM_001270584 | IQ motif containing with AAA domain 1 |
| 107 | MT1CP | 0.350 | OTTHUMT00000434321 | metallothionein 1C, pseudogene |
| 108 | SERPINB13 | 0.347 | NM_001307923 | serpin peptidase inhibitor, clade B (ovalbumin), member 13 |
| 109 | A2ML1 | 0.346 | NM_001282424 | alpha-2-macroglobulin-like 1 |
| 110 | ROS1 | 0.346 | NM_002944 | ROS proto-oncogene 1 , receptor tyrosine kinase |
| 111 | SNAR-B2 | 0.345 | NR_024230 | small ILF3/NF90-associated RNA B2 |
| 112 | SNAR-B2 | 0.345 | NR_024230 | small ILF3/NF90-associated RNA B2 |
| 113 | SNORD116-30 | 0.344 | NR_047032 | small nucleolar RNA, C/D box 116-30 |
| 114 | LOC105373853 | 0.344 | XR_923811 | uncharacterized LOC105373853 |
| 115 | SNORD116-21 | 0.341 | NR_003335 | small nucleolar RNA, C/D box 116-21 |
| 116 | SGK1 | 0.340 | NM_001143676 | serum/glucocorticoid regulated kinase 1 |
| 117 | LOC401585 | 0.340 | NR_125365 | uncharacterized LOC401585 |
| 118 | SNORD14A | 0.338 | NR_000022 | small nucleolar RNA, C/D box 14A |
| 119 | ZNF681 | 0.338 | NM_138286 | zinc finger protein 681 |
| 120 | TMEM98 | 0.337 | NM_001033504 | transmembrane protein 98 |
| 121 | NSUN5P1 | 0.336 | NR_033322 | NOP2/Sun domain family, member 5 pseudogene 1 |
| 122 | C10orf10 | 0.335 | NM_007021 | chromosome 10 open reading frame 10 |
| 123 | EGOT | 0.334 | NR_004428 | eosinophil granule ontogeny transcript (non-protein coding) |
| 124 | LOC105369669 | 0.333 | XM_011508340 | uncharacterized LOC105369669 |
| 125 | KYNU | 0.332 | NM_001032998 | kynureninase |
| 126 | SERPINB4 | 0.330 | NM_002974 | serpin peptidase inhibitor, clade B (ovalbumin), member 4 |
| 127 | AMIGO2 | 0.329 | NM_001143668 | adhesion molecule with Ig-like domain 2 |
| 128 | SNORD71 | 0.329 | NR_003059 | small nucleolar RNA, C/D box 71 |
| 129 | SNORD66 | 0.328 | NR_003055 | small nucleolar RNA, C/D box 66 |
| 130 | LOC105376374 | 0.328 | XR_930597 | uncharacterized LOC105376374 |
| 131 | MIR221 | 0.328 | NR_029635 | microRNA 221 |
| 132 | DAB2 | 0.326 | NM_001244871 | Dab, mitogen-responsive phosphoprotein, homolog 2 (Drosophila) |
| 133 | FCHSD1 | 0.322 | NM_033449 | FCH and double SH3 domains 1 |
| 134 | SNORD115-25 | 0.322 | NR_003342 | small nucleolar RNA, C/D box 115-25 |
| 135 | SNORD114-26 | 0.321 | NR_003219 | small nucleolar RNA, C/D box 114-26 |
| 136 | LOC101929988 | 0.320 | XR_916194 | uncharacterized LOC101929988 |
| 137 | DDR1 | 0.316 | NM_001297652 | discoidin domain receptor tyrosine kinase 1 |
| 138 | LOC101927841 | 0.315 | XR_252181 | uncharacterized LOC101927841 |
| 139 | ZNF675 | 0.314 | NM_138330 | zinc finger protein 675 |
| 140 | HSPA8 | 0.313 | NM_006597 | heat shock 70kDa protein 8 |
| 141 | SNORD104 | 0.312 | NR_004380 | small nucleolar RNA, C/D box 104 |
| 142 | MMP13 | 0.310 | NM_002427 | matrix metallopeptidase 13 |
| 143 | INHBA | 0.307 | NM_002192 | inhibin beta A |
| 144 | KIF3C | 0.307 | NM_002254 | kinesin family member 3C |
| 145 | UBC | 0.305 | NM_021009 | ubiquitin C |
| 146 | ITGB6 | 0.304 | NM_000888 | integrin beta 6 |
| 147 | TRAF1 | 0.304 | NM_001190945 | TNF receptor-associated factor 1 |
| 148 | FGF5 | 0.303 | NM_001291812 | fibroblast growth factor 5 |
| 149 | FAP | 0.300 | NM_001291807 | fibroblast activation protein alpha |
| 150 | SNAR-E | 0.298 | NR_024258 | small ILF3/NF90-associated RNA E |
| 151 | LOC101928100 | 0.296 | NR_120430 | uncharacterized LOC101928100 |
| 152 | LOC105369807 | 0.294 | XR_914193 | uncharacterized LOC105369807 |
| 153 | C1orf74 | 0.294 | NM_152485 | chromosome 1 open reading frame 74 |
| 154 | SNORD116-29 | 0.293 | NR_003360 | small nucleolar RNA, C/D box 116-29 |
| 155 | SNORA75 | 0.292 | NR_002921 | small nucleolar RNA, H/ACA box 75 |
| 156 | SNORD59A | 0.289 | NR_002737 | small nucleolar RNA, C/D box 59A |
| 157 | SNORA14B | 0.288 | NR_002956 | small nucleolar RNA, H/ACA box 14B |
| 158 | SNORD116-18 | 0.287 | NR_003333 | small nucleolar RNA, C/D box 116-18 |
| 159 | CXCL8 | 0.287 | NM_000584 | chemokine (C-X-C motif) ligand 8 |
| 160 | MIR590 | 0.284 | NR_030321 | microRNA 590 |
| 161 | LINC-PINT | 0.284 | NR_034120 | long intergenic non-protein coding RNA, p53 induced transcript |
| 162 | IL36G | 0.283 | NM_001278568 | interleukin 36, gamma |
| 163 | NEAT1 | 0.281 | NR_131012 | nuclear paraspeckle assembly transcript 1 (non-protein coding) |
| 164 | ZNF91 | 0.279 | NM_001300951 | zinc finger protein 91 |
| 165 | SNORD114-15 | 0.279 | NR_003208 | small nucleolar RNA, C/D box 114-15 |
| 166 | SNORD114-20 | 0.275 | NR_003213 | small nucleolar RNA, C/D box 114-20 |
| 167 | SCEL | 0.274 | NM_001160706 | sciellin |
| 168 | THBS1 | 0.274 | NM_003246 | thrombospondin 1 |
| 169 | VCAN | 0.274 | NM_001126336 | versican |
| 170 | SNORD99 | 0.272 | NR_003077 | small nucleolar RNA, C/D box 99 |
| 171 | MIR21 | 0.272 | NR_029493 | microRNA 21 |
| 172 | SLC46A3 | 0.271 | NM_001135919 | solute carrier family 46, member 3 |
| 173 | KRT6C | 0.270 | NM_173086 | keratin 6C, type II |
| 174 | SNORA60 | 0.269 | NR_002986 | small nucleolar RNA, H/ACA box 60 |
| 175 | RUNX1-IT1 | 0.266 | NR_026812 | RUNX1 intronic transcript 1 |
| 176 | KCNJ15 | 0.266 | NM_001276435 | potassium channel, inwardly rectifying subfamily J, member 15 |
| 177 | RPS2 | 0.265 | ENST00000343262 | ribosomal protein S2 |
| 178 | CXCL11 | 0.264 | NM_001302123 | chemokine (C-X-C motif) ligand 11 |
| 179 | LOC100506895 | 0.262 | NR_038276 | uncharacterized LOC100506895 |
| 180 | LOC100506123 | 0.261 | uc021vll.1 | uncharacterized LOC100506123 |
| 181 | NLRP10 | 0.258 | NM_176821 | NLR family, pyrin domain containing 10 |
| 182 | RNF152 | 0.257 | NM_173557 | ring finger protein 152 |
| 183 | LOC100130476 | 0.254 | NR_049793 | uncharacterized LOC100130476 |
| 184 | SNORD114-21 | 0.253 | NR_003214 | small nucleolar RNA, C/D box 114-21 |
| 185 | TGM2 | 0.253 | NM_004613 | transglutaminase 2 |
| 186 | MEG3 | 0.252 | NR_002766 | maternally expressed 3 (non-protein coding) |
| 187 | KRTAP19-1 | 0.249 | ENST00000390689 | keratin associated protein 19-1 |
| 188 | TNFAIP3 | 0.249 | NM_001270507 | tumor necrosis factor, alpha-induced protein 3 |
| 189 | CTGF | 0.248 | NM_001901 | connective tissue growth factor |
| 190 | S100A12 | 0.247 | NM_005621 | S100 calcium binding protein A12 |
| 191 | SPRR3 | 0.247 | NM_001097589 | small proline-rich protein 3 |
| 192 | IGFL1 | 0.247 | NM_198541 | IGF like family member 1 |
| 193 | MMP12 | 0.246 | NM_002426 | matrix metallopeptidase 12 |
| 194 | SNORD92 | 0.246 | NR_003074 | small nucleolar RNA, C/D box 92 |
| 195 | CSF2 | 0.243 | NM_000758 | colony stimulating factor 2 (granulocyte-macrophage) |
| 196 | KLK6 | 0.242 | NM_001012964 | kallikrein related peptidase 6 |
| 197 | DEFB103A | 0.239 | NM_001081551 | defensin, beta 103A |
| 198 | DEFB103A | 0.239 | NM_001081551 | defensin, beta 103A |
| 199 | SNORD116-1 | 0.238 | NR_003316 | small nucleolar RNA, C/D box 116-1 |
| 200 | LINC00862 | 0.237 | NR_040064 | long intergenic non-protein coding RNA 862 |
| 201 | GANC | 0.236 | ENST00000318010 | glucosidase, alpha; neutral C |
| 202 | SNORD114-9 | 0.235 | NR_003201 | small nucleolar RNA, C/D box 114-9 |
| 203 | CXCL1 | 0.233 | NM_001511 | chemokine (C-X-C motif) ligand 1 (melanoma growth stimulating activity, alpha) |
| 204 | LYPD5 | 0.233 | NM_001031749 | LY6/PLAUR domain containing 5 |
| 205 | HNRNPA1P33 | 0.230 | NR_003277 | heterogeneous nuclear ribonucleoprotein A1 pseudogene 33 |
| 206 | SNORD12C | 0.228 | NR_002433 | small nucleolar RNA, C/D box 12C |
| 207 | SPRR1A | 0.218 | NM_001199828 | small proline-rich protein 1A |
| 208 | SNORD115-17 | 0.213 | NR_003309 | small nucleolar RNA, C/D box 115-17 |
| 209 | SNORD115-17 | 0.213 | NR_003309 | small nucleolar RNA, C/D box 115-17 |
| 210 | SNORD115-17 | 0.213 | NR_003309 | small nucleolar RNA, C/D box 115-17 |
| 211 | SNORD115-1 | 0.213 | NR_001291 | small nucleolar RNA, C/D box 115-1 |
| 212 | SNORD115-16 | 0.213 | NR_003308 | small nucleolar RNA, C/D box 115-16 |
| 213 | SNORD18B | 0.212 | NR_002442 | small nucleolar RNA, C/D box 18B |
| 214 | CXCL5 | 0.209 | NM_002994 | chemokine (C-X-C motif) ligand 5 |
| 215 | SCARNA17 | 0.209 | NR_003003 | small Cajal body-specific RNA 17 |
| 216 | HEPHL1 | 0.208 | NM_001098672 | hephaestin-like 1 |
| 217 | MRGPRX3 | 0.206 | NM_054031 | MAS-related GPR, member X3 |
| 218 | SLC6A14 | 0.206 | NM_007231 | solute carrier family 6 (amino acid transporter), member 14 |
| 219 | CPA4 | 0.204 | NM_001163446 | carboxypeptidase A4 |
| 220 | SNORD115-27 | 0.205 | NR_003496 | small nucleolar RNA, C/D box 115-27 |
| 221 | NEFM | 0.202 | NM_001105541 | neurofilament, medium polypeptide |
| 222 | SNORD114-12 | 0.201 | NR_003205 | small nucleolar RNA, C/D box 114-12 |
| 223 | IL6 | 0.199 | NM_000600 | interleukin 6 |
| 224 | LCE1F | 0.194 | NM_178354 | late cornified envelope 1F |
| 225 | SNORD115-30 | 0.192 | NR_003345 | small nucleolar RNA, C/D box 115-30 |
| 226 | ZNF117 | 0.189 | NM_015852 | zinc finger protein 117 |
| 227 | FLG2 | 0.188 | NM_001014342 | filaggrin family member 2 |
| 228 | LCE6A | 0.186 | NM_001128600 | late cornified envelope 6A |
| 229 | SPRR2B | 0.182 | NM_001017418 | small proline-rich protein 2B |
| 230 | NEFL | 0.178 | NM_006158 | neurofilament, light polypeptide |
| 231 | C4orf26 | 0.178 | NM_001206981 | chromosome 4 open reading frame 26 |
| 232 | SPINK5 | 0.177 | NM_001127698 | serine peptidase inhibitor, Kazal type 5 |
| 233 | CEACAM6 | 0.176 | NM_002483 | carcinoembryonic antigen-related cell adhesion molecule 6 (non-specific cross reacting antigen) |
| 234 | KRT1 | 0.175 | NM_006121 | keratin 1, type II |
| 235 | AMTN | 0.175 | NM_001286731 | amelotin |
| 236 | ENC1 | 0.175 | NM_001256574 | ectodermal-neural cortex 1 (with BTB domain) |
| 237 | SNORD115-20 | 0.170 | NR_003312 | small nucleolar RNA, C/D box 115-20 |
| 238 | SNORD114-28 | 0.167 | NR_003221 | small nucleolar RNA, C/D box 114-28 |
| 239 | ICAM1 | 0.164 | NM_000201 | intercellular adhesion molecule 1 |
| 240 | KRT34 | 0.163 | NM_021013 | keratin 34, type I |
| 241 | SCARNA7 | 0.162 | NR_003001 | small Cajal body-specific RNA 7 |
| 242 | MUCL1 | 0.161 | NM_058173 | mucin-like 1 |
| 243 | SNORD115-39 | 0.160 | NR_003354 | small nucleolar RNA, C/D box 115-39 |
| 244 | FLG | 0.159 | NM_002016 | filaggrin |
| 245 | SNORD115-44 | 0.156 | NR_003359 | small nucleolar RNA, C/D box 115-44 |
| 246 | HIST1H2BG | 0.155 | NM_003518 | histone cluster 1, H2bg |
| 247 | IL24 | 0.147 | NM_001185156 | interleukin 24 |
| 248 | LCE1A | 0.145 | NM_178348 | late cornified envelope 1A |
| 249 | LOC105374003 | 0.144 | XR_924265 | uncharacterized LOC105374003 |
| 250 | SNORD93 | 0.144 | NR_003075 | small nucleolar RNA, C/D box 93 |
| 251 | KPRP | 0.139 | NM_001025231 | keratinocyte proline-rich protein |
| 252 | BEX1 | 0.139 | NM_018476 | brain expressed X-linked 1 |
| 253 | SPRR4 | 0.133 | NM_173080 | small proline-rich protein 4 |
| 254 | NCCRP1 | 0.128 | NM_001001414 | non-specific cytotoxic cell receptor protein 1 homolog (zebrafish) |
| 255 | SNORD115-22 | 0.128 | NR_003314 | small nucleolar RNA, C/D box 115-22 |
| 256 | SNORD115-5 | 0.127 | NR_003297 | small nucleolar RNA, C/D box 115-5 |
| 257 | SNORD115-9 | 0.127 | NR_003301 | small nucleolar RNA, C/D box 115-9 |
| 258 | SNORD115-11 | 0.127 | NR_003303 | small nucleolar RNA, C/D box 115-11 |
| 259 | SNORD115-9 | 0.127 | NR_003301 | small nucleolar RNA, C/D box 115-9 |
| 260 | SNORD115-11 | 0.127 | NR_003303 | small nucleolar RNA, C/D box 115-11 |
| 261 | SNORD115-11 | 0.127 | NR_003303 | small nucleolar RNA, C/D box 115-11 |
| 262 | SNORD115-11 | 0.127 | NR_003303 | small nucleolar RNA, C/D box 115-11 |
| 263 | LOC101929260 | 0.126 | NR_110241 | uncharacterized LOC101929260 |
| 264 | SNORD115-6 | 0.125 | NR_003298 | small nucleolar RNA, C/D box 115-6 |
| 265 | SNORD115-42 | 0.125 | NR_003357 | small nucleolar RNA, C/D box 115-42 |
| 266 | CXCL10 | 0.121 | NM_001565 | chemokine (C-X-C motif) ligand 10 |
| 267 | LOC105376382 | 0.114 | XR_930613 | uncharacterized LOC105376382 |
| 268 | RPTN | 0.098 | NM_001122965 | repetin |
| 269 | SNORD114-3 | 0.096 | NR_003195 | small nucleolar RNA, C/D box 114-3 |
| 270 | CNFN | 0.073 | NM_032488 | cornifelin |
| 271 | SPRR2G | 0.071 | NM_001014291 | small proline-rich protein 2G |
| 272 | SNORD114-1 | 0.071 | NR_003193 | small nucleolar RNA, C/D box 114-1 |
| 273 | PI3 | 0.065 | NM_002638 | peptidase inhibitor 3, skin-derived |
| 274 | S100A7 | 0.050 | NM_002963 | S100 calcium binding protein A7 |
| 275 | KRTAP2-3 | 0.049 | NM_001165252 | keratin associated protein 2-3 |
| 276 | CRCT1 | 0.039 | NM_019060 | cysteine rich C-terminal 1 |
| 277 | LCE3E | 0.034 | NM_178435 | late cornified envelope 3E |
| 278 | LCE3D | 0.012 | NM_032563 | late cornified envelope 3D |

* The value applies the fold change which compares short-term and long-term cultured samples. It is averaged between two individual samples that were separately experimented.
